# Involvement of Microtubular Network and Its Motors in Productive Endocytic Trafficking of Mouse Polyomavirus

**DOI:** 10.1371/journal.pone.0096922

**Published:** 2014-05-08

**Authors:** Vojtech Zila, Francesco Difato, Lucie Klimova, Sandra Huerfano, Jitka Forstova

**Affiliations:** Department of Genetics and Microbiology, Faculty of Science, Charles University in Prague, Prague, Czech Republic; CNRS, France

## Abstract

Infection of non-enveloped polyomaviruses depends on an intact microtubular network. Here we focus on mouse polyomavirus (MPyV). We show that the dynamics of MPyV cytoplasmic transport reflects the characteristics of microtubular motor-driven transport with bi-directional saltatory movements. In cells treated with microtubule-disrupting agents, localization of MPyV was significantly perturbed, the virus was retained at the cell periphery, mostly within membrane structures resembling multicaveolar complexes, and at later times post-infection, only a fraction of the virus was found in Rab7-positive endosomes and multivesicular bodies. Inhibition of cytoplasmic dynein-based motility by overexpression of dynamitin affected perinuclear translocation of the virus, delivery of virions to the ER and substantially reduced the numbers of infected cells, while overexpression of dominant-negative form of kinesin-1 or kinesin-2 had no significant impact on virus localization and infectivity. We also found that transport along microtubules was important for MPyV-containing endosome sequential acquisition of Rab5, Rab7 and Rab11 GTPases. However, in contrast to dominant-negative mutant of Rab7 (T22N), overexpression of dominant-negative mutant Rab11 (S25N) did not affect the virus infectivity. Altogether, our study revealed that MPyV cytoplasmic trafficking leading to productive infection bypasses recycling endosomes, does not require the function of kinesin-1 and kinesin-2, but depends on functional dynein-mediated transport along microtubules for translocation of the virions from peripheral, often caveolin-positive compartments to late endosomes and ER – a prerequisite for efficient delivery of the viral genome to the nucleus.

## Introduction

Mouse polyomavirus (MPyV) is a tumor virus belonging to the *Polyomaviridae* family, whose members are small non-enveloped DNA viruses replicating in the cell nucleus. MPyV enter cells by clathrin- and caveolae-independent endocytosis [1]–[3], through the interaction of the major viral capsid protein, VP1, with ganglioside receptors, GD1a or GT1b [4] that associate with lipid rafts [5], [6]. Efficient uptake of MPyV also requires remodeling of the actin cytoskeleton [2], [5], [7]. Virions are internalized into smooth endocytic vesicles [3], [6], [8], often positive for caveolin-1 [5], [6], and subsequently fuse with larger endosomes [3], [5], [6], [9]. Like other polyomaviruses, MPyV virions do not escape the endosomal system until they reach the lumen of smooth endoplasmic reticulum (ER) [5], [6], [9], [10], where lumenal enzymes facilitate virus capsid disassembly and uncoating of viral genomes prior to their import into the nucleus [11]–[13]. Regardless of the multiplicity of infection, only a few virions are able to deliver their genomic DNA into the cell nucleus [10]. Currently, two possible ways for viral genome delivery to the cell nucleus have been proposed: either partially disassembled virions translocate from ER to the cytosol and then are imported into the nucleus via nuclear pore complexes or, alternatively, they penetrate directly from ER to the nucleoplasm through the nuclear envelope (reviewed in [14]).

Infection of MPyV is dependent on an intact microtubular network and microtubule depolymerizing drugs such as nocodazole or colcemid block virus infectivity [2], [5], [15], [16]. The virus infectious pathway to ER is directed from the plasma membrane by the ganglioside receptor [9], [17] and requires acidic environment of the endosomes [3], [9]. Previous reports pointed to the importance of virus transfer via early and late endosomal compartments as overexpression of dominant-negative mutant of Rab5 [3], [9] or Rab7 GTPase [9] inhibited MPyV infection. On the other hand, caveolin-1, accompanying the internalization and trafficking of the subpopulation of MPyV virions towards the nucleus, is not required for virus infectivity [3]. Our previous studies reported the virus presence in perinuclear area of cells co-localizing there with Rab11 GTPase and with transferrin, markers of recycling endosomes [3], [10], suggesting the possibility that transfer of MPyV via mildly acidic early endosomes and later via recycling endosomes represents an alternative endocytic pathway along microtubules that virions can use for genome delivery into the host cell nucleus.

Intact microtubules are also essential for other polyomaviruses, including simian virus 40 (SV40), or human polyomaviruses BK virus (BKV) or JC virus (JCV) [18]–[24]. Although in some cells the trafficking of SV40 occurs in vesicles propelled by actin polymerization, functional microtubule-dependent transport was found to be essential for delivery of SV40 virions from acidic late endosomes to ER [23], [25]. Similarly, involvement of microtubules in virus transport from acidic endosomes to the ER has been proposed for BKV [24]. It has been shown that microtubules mediate trafficking of MPyV from ‘caveolin-1-positive environment’ to the ER [5]. Authors of this study suggested that caveolin-1-rich compartments may represent so-called “caveosomes”. However, since the caveosomes defined as caveolin-1-positive, pH-neutral organelles were recently identified as modified late endosomes or lysosomes [23], [26], the involvement of microtubular network in endocytic trafficking of MPyV to early and premature late endosomes is not clear.

The requirement of intact microtubules for MPyV infection implies that specific microtubule-associated motor(s) play a key role in the intracellular trafficking of the virus, but these kinetic aspects of the MPyV infection pathway still remain to be determined. Microtubule-associated motors are essential for the function and spatio-temporal organization of the endosomal system. Opposing forces provided by dynein and kinesins are responsible for the oscillatory motion of endosomes along microtubules and are required for endosome trafficking towards the nucleus, plasma membrane or vesicular transport between other membrane compartments (reviewed in [27]). In contrast to JCV- and BKV-infected cells, where inhibition of dynein has an insignificant effect on virus infectivity [18], [20], SV40 relies on functional dynein for infection at least in HeLa cells [23]. The possible role of kinesin-1 motor, which together with dynein is utilized by some other viruses for transport to the proximity of the nucleus (reviewed in [28]), or kinesin-2 motor, which together with dynein and kinesin-1 has been shown to be important for the motility of early and late endosomes (reviewed in [29]), has not yet been investigated for any polyomavirus.

In this study, we used confocal fluorescence microscopy of living cells to examine the dynamics of MPyV cytoplasmic trafficking. Further, we performed infectivity assays to test the effect of dominant-negative forms of dynein, kinesin-1 or kinesin-2 motor on MPyV productive transport and we used confocal and electron microscopy approaches to monitoring the involvement of microtubular network in transport of MPyV from the plasma membrane to classical endosomes and in subsequent virus delivery to the ER. Finally, we investigated whether virus transfer via recycling endosomes represents an alternative endocytic pathway along microtubules utilized by MPyV to reach the cell nucleus.

## Results

### MPyV Trafficking along Microtubular Tracks

**Figure 1:**
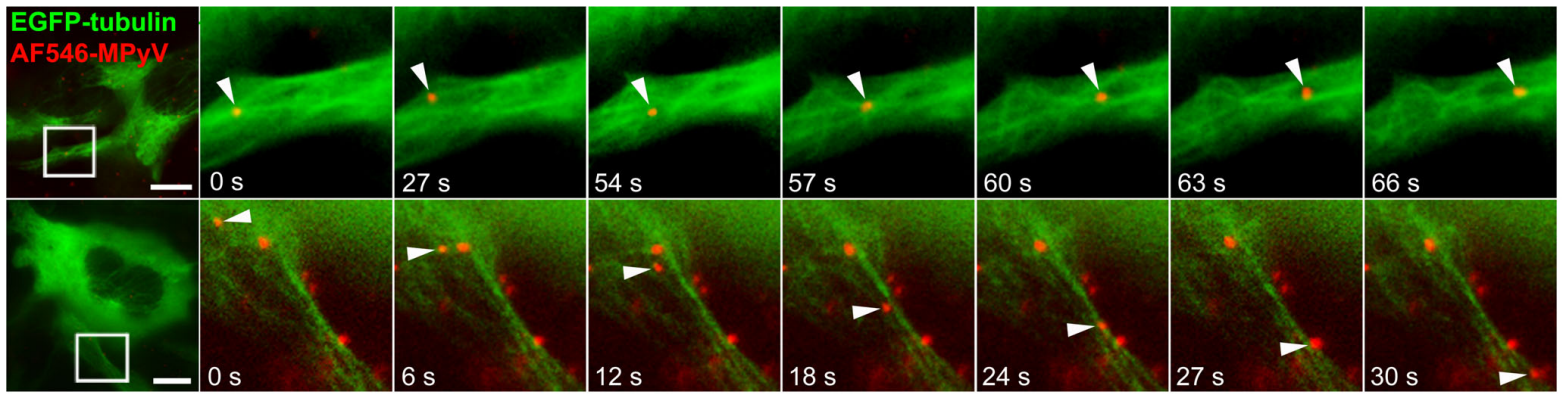
Tracking of MPyV in living cells expressing EGFP-fused tubulin. 3T6 cells expressing EGFP-tubulin (green) were infected with Alexa Fluor 546-labeled MPyV (red) (MOI of 10^2^ to 10^3^ virus particles per cell) at 37°C and scanned with ΔT = 3 s. Virions were transported to both directions: to the nuclear periphery (upper panel) and to the cell periphery (lower panel). Selected frames from two different cells at 1 h p.i. are shown in detail (see Movie S1). Arrowheads point to MPyV virions. Bars, 10 µm. Cells were examined with an Olympus IX81 CellR microscope equipped with an MT20 illumination system.

We prepared a stable cell line of mouse 3T6 fibroblasts expressing α-tubulin fused with enhanced green fluorescent protein (EGFP) to follow the direct involvement of microtubules during MPyV trafficking in live cells. Cells expressing EGFP-tubulin were infected with fluorescently labeled MPyV and transport of virions along microtubules was followed at early stages (up to 2 h) post-infection (p.i.) by time-lapse fluorescence microscopy. We observed virus transport along microtubules soon after virus addition (20–30 min p.i.). Virions were moved along microtubules in a bi-directional manner, being transported into the cell interior but also back to the cell periphery (Figure 1, Movie S1). Although the virus cargo was transported in both directions (often along an identical microtubule), the virus fluorescent signal was found accumulated around the nucleus at later times post-infection (from ∼3 h p.i.) (Movie S2). This observation indicates that MPyV transport to the vicinity of the nucleus is prevalent during a longer time span of trafficking.

### Dynamics of MPyV Cytoplasmic Transport by Single Particle Tracking

**Figure 2:**
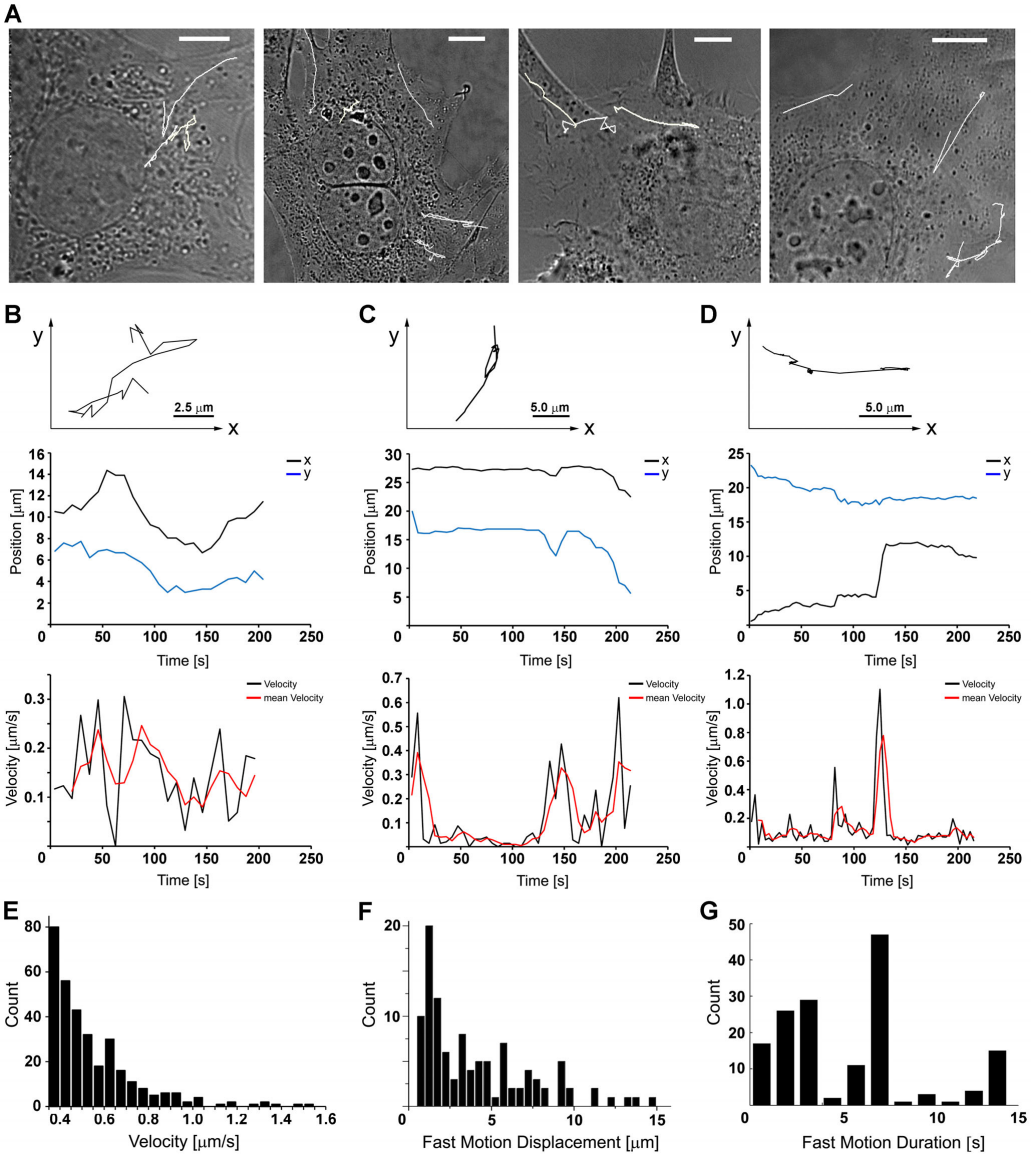
Single particle tracking analysis of MPyV transport. 3T6 cells were infected with Alexa Fluor 546-labeled MPyV (MOI of 10^2^ to 10^3^ virus particles per cell) at 37°C and scanned with ΔT = 6 s. (A) Complex trajectories marked in white tracking curves in four selected cells shown in transmission light. Bars, 5 µm. (B–D) Dynamics of three independent single particle trackings (upper graphs) representing three main types of virion transport velocities with single particle position tracking in x-y coordinates (middle graphs) and course of movement velocities (lower graphs) measured at time intervals of 6 s (mean velocity was averaged from each three following time steps). (E) Frequency of virion transport velocity rates, counted from more than 200 different tracking experiments in 3T6 cells, with a distinct peak at 0.6 µm/s (x-coordinate was cropped to cut off the high frequency of short-range movements at rates <0.3 µm/s). (F) Histogram of fast movement distances counted from 109 single fast movements and sampled into 0.5 µm step intervals, with a maximum at 1.5 µm and the average distance of 2.5 µm. (G) Time span frequency of fast (≥0.6 µm/s) movements with maximum counts at 3.3 and 7.2 s.

To describe the dynamics of the MPyV trafficking, we performed single particle tracking analysis and mapped transport trajectories of fluorescently labeled virions in living 3T6 cells. In order to monitor the virus intracellular movement from infection start until the time when the virus appears in the proximity of the nucleus, we followed trafficking of virions from 30 min to 3 h p.i. As a control we performed the internalization assay, which showed that a considerable amount of virions (∼50%) was already internalized after 30 min of infection and that the major part of the virus was internalized at 60 min p.i. (Figure S1). The presence of virions attached to the cell surface was taken into consideration during the virus tracking experiments (see Materials and Methods). Virus trajectories revealed complex patterns. They were not clearly oriented from the cell surface towards the nucleus, but instead, they displayed the characteristics of Brownian motion with fast random switching in velocities and direction of movement (Figure 2A). The dynamics of virion transport was mostly saltatory – the fast forward movements were interrupted by short back-step movements or pausing at intervals (average pause duration of 40±6.5 s). Our measurements of movement velocities corresponded to approximately three main characteristic rates (trajectories and graphs in Figure 2B,C,D): i) slow movement (in a limited space) at rates up to 0.3 µm/s, ii) faster movement of virions with frequent velocity rate around 0.6 µm/s, and iii) fast, long-distance movement with peaks reaching rates of 1.2 µm/s – speed typical of motor-driven transport on microtubular tracks [30].

Following movements of more than 200 single virions in about 40 distinct cells, we computed the frequency of velocity rates. The most prevalent were slow movements (<0.2 µm/s) (Figure 2E), which can be assigned to pausing of virus-loaded vesicles on microtubule tracks before another motor was recruited (or activated), or to actin-driven motility of virus-carrying endosomes as the velocity of vesicles/endosomes propelled by actin polymerization reaches rates around 0.2 µm/s [31]–[33]. The association of slowly moved MPyV-loaded endosomes with assemblies of dynamic actin recruited at their membranes was confirmed in living EGFP-actin-expressing cells (Figure S2, Movie S3). During such a “stationary phase”, we detected movement only in a limited range (0.5–1 µm). In contrast, fast, long-distance movements (up to 1.5 µm/s) were rare, with a distinct peak at 0.6 µm/s (Figure 2E). Our measurements of fast movements (≥0.6 µm/s) revealed that the average length of a single continuous movement was 2.5 µm (Figure 2F). Such values could correspond to recruitment of a single microtubular motor molecule on the transported vesicle and fit well with the processivity of individual kinesin or dynein molecule-mediated transport producing movements of approximately 1.5 µm before the motor dissociates from the microtubule [34]. We observed that events of faster (>0.6 µm/s) long-range transport occurred exclusively for short time intervals and the time span of the movements was characterized by peaks at 3.3 and 7.2 s (Figure 2G). These data indicate that in the case of MPyV, the run length of the vesicle carrying the virus cargo-motor complex reflected an inherent microtubular motor processivity.

### Dynein Motor is Essential for MPyV Infection and Virus Trafficking to the ER

**Figure 3:**
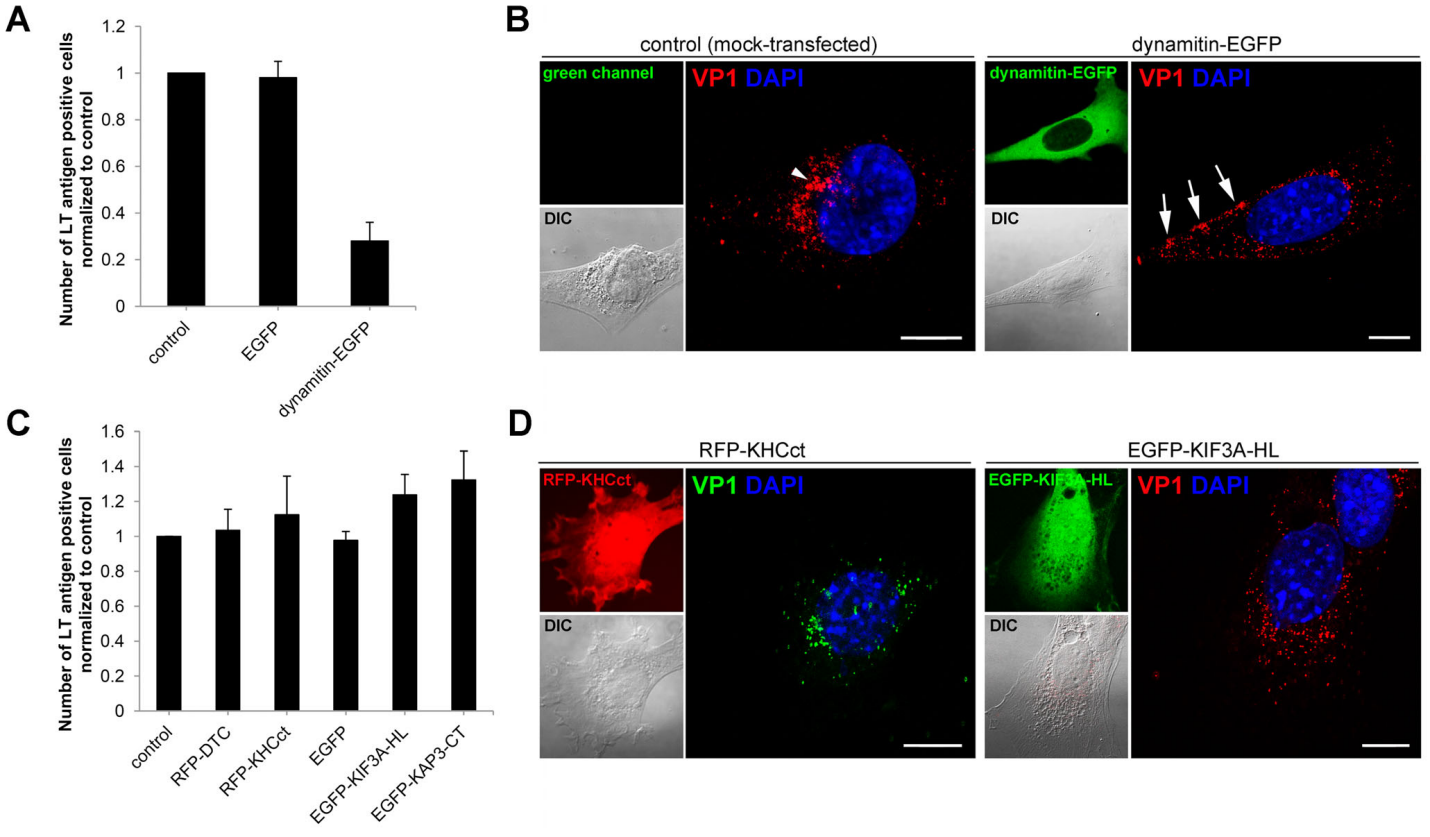
Role of microtubular motors in MPyV productive trafficking. (A and C) 3T6 cells were transfected with: (A) plasmid DNA for transient expression of EGFP (pEGFP-N1) or dynamitin-EGFP (inhibiting the dynein motor function) or (C) plasmid DNA for transient expression of RFP-DTC protein (pRFP-DTC), RFP-fused C-terminal fragment of kinesin-1 (pRFP-KHCct; inhibiting kinesin-1 motor function), EGFP (pEGFP-N1) or EGFP-fused dominant-negative subunits of kinesin-2 (pEGFP-KIF3A-HL or pEGFP-KAP3-CT). Cells expressing constructs were infected with MPyV, incubated until 24 h p.i., fixed and immunostained for MPyV LT antigen. The efficiency of infection was determined by levels (%) of LT antigen-positive cells normalized to that obtained in control, mock-transfected cells. During the experiment, more than 500 cells were counted for each sample. Data in the graphs represent mean values ± s.d. from three independent experiments. (B and D) Control, mock-transfected cells, or cells expressing dynamitin-EGFP, RFP-KHCct or EGFP-KIF3A-HL, infected with MPyV (MOI of 10^3^ virus particles per cell), fixed 5 h p.i. and immunostained for MPyV VP1 capsid protein. DNA in nuclei was stained with DAPI (blue). Confocal sections of representative cells with corresponding signal in green or red channel and differential interference contrast (DIC) images are presented (the virus localization during EGFP-KIF3A-CT expression is not presented as it was similar to that in cells expressing the -HL form of kinesin-2). Arrowhead point to the virus at nuclear periphery. Arrows point to the virions at cell periphery. Bars, 10 µm.

The observation of the bi-directionality and dynamics of long-distance movements of single MPyV virions suggested the possible involvement of plus end-oriented kinesins and minus end-oriented dynein during virus endocytic trafficking towards the nucleus. We therefore investigated the role of dynein, kinesin-1 and kinesin-2 motor in productive trafficking of MPyV. The importance of the dynein motor for MPyV infection was tested by infectivity assays in 3T6 cells transiently overexpressing EGFP-fused dynamitin, as overexpression of dynamitin causes disassembly of the dynein-dynactin complex and thus inhibits dynein motor function [35]–[37]. The efficiency of infection of these cells was compared with that of control, mock-transfected cells and cells expressing EGFP alone. Compared to controls, overexpression of EGFP-dynamitin dramatically reduced the number of infected cells (more than 70% decrease) (Figure 3A). When confocal fluorescence microscopy was performed later (5 h) post-infection in dynamitin-EGFP expressing cells, substantial amounts of virions were still detected at the cell periphery (Figure 3B, right panel), while in mock-transfected cells, the virus was accumulated predominantly in perinuclear area (Figure 3B, left panel). To investigate the importance of kinesin-1 and kinesin-2 motor for MPyV infection, we performed infectivity assays in cells expressing their dominant-negative constructs. The function of kinesin-1 was inhibited by overexpression of the C-terminal domain of kinesin-1 heavy chain fused with red fluorescent protein (RFP-KHCct), as the expression of C-terminal segment inhibits kinesin-1-driven microtubule activity by binding to the kinesin motor domain [38]–[40]. The kinesin-2 motor was inhibited by overexpression of EGFP-fused dominant-negative subunits of kinesin-2, motorless KIF3A subunit (EGFP-KIF3A-HL), or C-terminus of KAP3 subunit (EGFP-KAP3-CT) [41], [42]. For control, we infected mock-transfected cells, cells expressing RFP-fused form of kinesin-1 light chain (RFP-DTC) as negative control for kinesin-1 [39], [40], and cells expressing EGFP alone as a control for EGFP-fused kinesin-2 constructs. Production of dominant-negative kinesin-1 or kinesin-2 did not reduce the virus infectivity. Moreover, a slight increase in the number of infected cells was detected when compared to controls (Figure 3C). Confocal microscopy of cells producing dominant-negative kinesin-1 or kinesin-2 performed at 5 h p.i. did not reveal any significant difference in virus localization when compared to mock-transfected cells (Figure 3D). Together, these results indicate that transport mediated by the dynein motor is critical for MPyV infection and its perinuclear sorting, whereas the function of kinesin-1 or kinesin-2 is dispensable.

**Figure 4:**
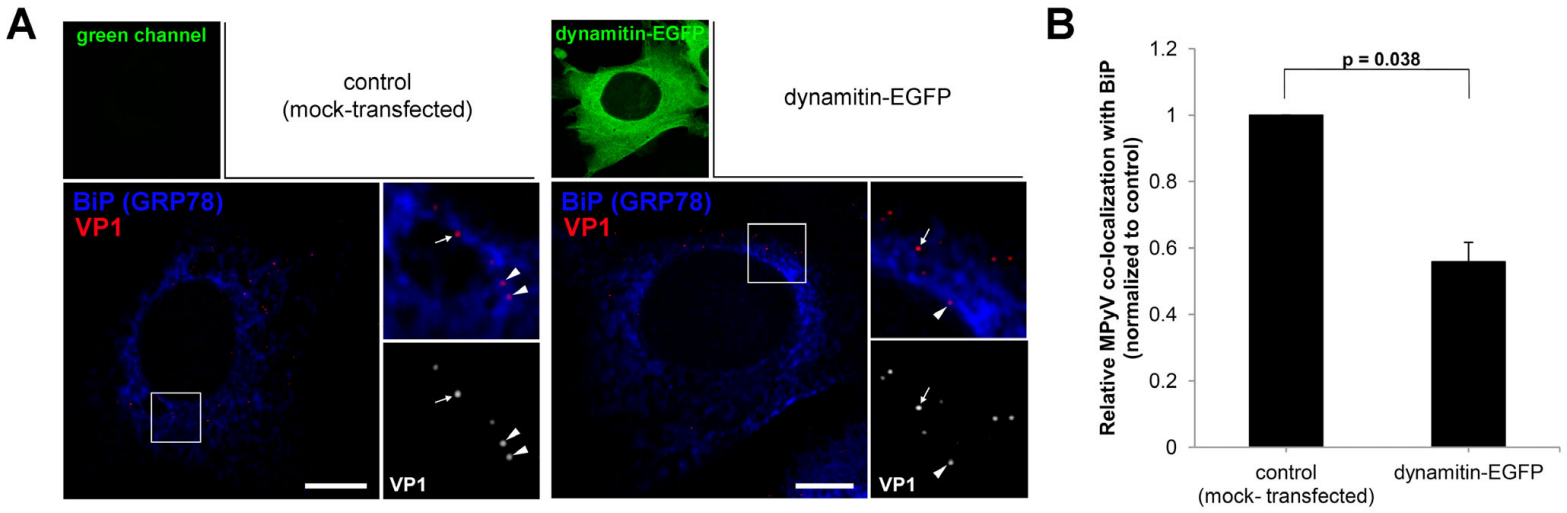
Dynein motor is required for trafficking of MPyV to the ER. 3T6 cells were transfected with plasmid DNA for expression of dynamitin-EGFP, infected with MPyV (MOI of 5×10^2^ virus particles per cell) and fixed 5 h p.i. Cells were immunostained for MPyV VP1 capsid protein (red) and BiP (GRP78) marker of ER (blue). (A) Confocal sections of representative control (mock-transfected) cells and dynamitin-EGFP expressing cells at 5 h p.i. with enlarged details. Arrowheads point to selected MPyV virions co-localized with BiP protein. Arrows point to selected MPyV that did not co-localize with BiP protein. Bars, 10 µm. (B) Quantification of co-localization of MPyV virions with BiP at 5 h p.i. The percentage of co-localizing virions was calculated from images such as shown in panel A and levels (%) of co-localizing virions in dynamitin-expressing cells were normalized to that in control. During the experiment, more than 600 virions in at least 10 different cells were evaluated for each sample. Data in the graph represent mean values ± s.d. from three independent experiments; Student’s t-test was used.

As we observed reduced MPyV infectivity in dynamitin-overexpressing cells, we next investigated the involvement of the dynein motor in delivery of MPyV virions to the ER. For this, we infected dynamitin-EGFP-expressing cells and fixed them 5 h p.i. In the cells we followed and quantified co-localization of individual MPyV virions with the BiP (GRP78) protein as a marker of ER and compared it with that in control, mock-transfected cells. Co-localization of the VP1 signal of MPyV virions with the fluorescent signal of BiP protein was quantified from confocal images such as those shown in Figure 4A, as explained in Materials and Methods. The quantification revealed that inhibition of the dynein motor function by overexpression of dynamitin significantly reduced virus co-localization with BiP (by 40–50%) when compared to that in control cells (Figure 4B). We thus conclude that the dynein motor function is essential for the delivery of MPyV to ER.

### Microtubules are Important for Trafficking of MPyV to Endosomes

Investigation of SV40 trafficking showed that transport of the virus to early and premature late endosomes is independent of intact microtubules and that microtubules are required later for maturation of SV40-carrying late endosomes and subsequent virus delivery to the ER [23]. However, the above-described effect of overexpression of dynamitin-EGFP on MPyV intracellular localization suggests that dynein-mediated transport is required soon after virus internalization for virus trafficking from the plasma membrane to endosomes. To dissect the involvement of microtubules or actin cytoskeleton in the presence of MPyV in individual endocytic compartments, we infected 3T6 cells (non-transfected or expressing EGFP-fused marker of interest) in the absence or presence of compounds selectively affecting the structure and dynamics of microtubules (nocodazole) or actin cytoskeleton (latrunculin A) and fixed them 5 h p.i. In the cells we followed and quantified tendencies in co-localization of individual MPyV virions with endocytic markers previously shown to be involved in trafficking of MPyV towards the nucleus: caveolin-1 for caveolar or other compartments functionally connected to lipid rafts, EGFP-Rab5 GTPase for early endosomes, EGFP-Rab7 GTPase for late endosomes, EGFP-Rab11 GTPase for recycling endosomes, and BiP protein as ER marker.

**Figure 5:**
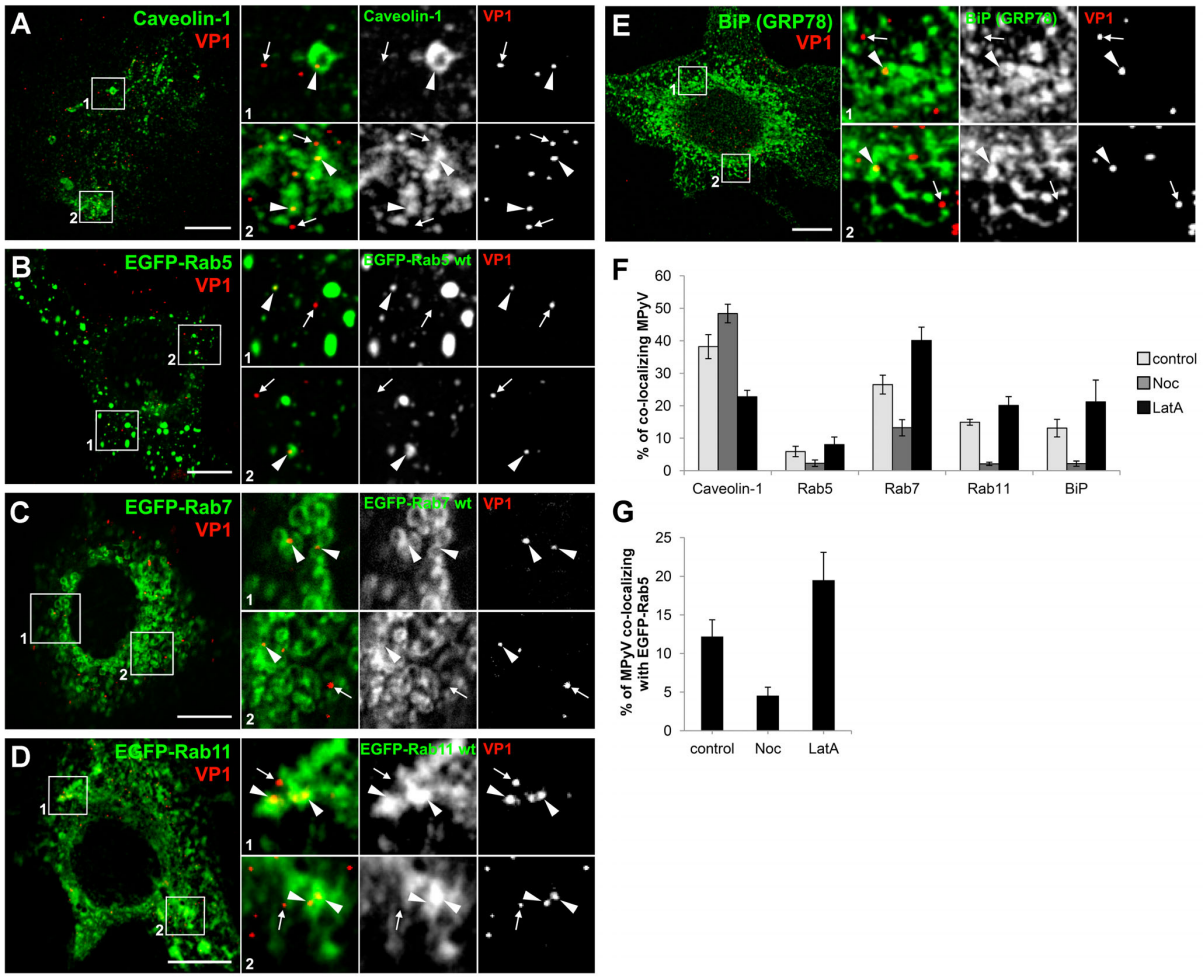
Effect of cytoskeleton-disrupting drugs on subcellular localization of MPyV. Non-transfected 3T6 cells (A and E) or cells transiently expressing EGFP-tagged marker of interest (B–D) were infected with MPyV (MOI of 5×10^2^ virus particles per cell) and fixed 5 h p.i. Cells were immunostained for MPyV VP1 capsid protein (red) and for a second marker of interest (caveolin-1, BiP) if not fused with EGFP (green). Confocal sections of cells with enlarged details are shown. Arrowheads point to selected MPyV virions co-localized with the marker of interest. Arrows point to selected MPyV that did not co-localize with the marker of interest. Bars, 10 µm. (F) Quantification of co-localization of MPyV virions with indicated markers at 5 h p.i. in non-treated cells (control) or cells pre-treated (1 h) and infected in the presence of nocodazole (Noc) or latrunculin A (LatA). (G) Quantification of co-localization of MPyV virions with EGFP-Rab5 at 1.5 h p.i., in control, Noc- or LatA-treated cells. The percentage of co-localizing virions was calculated from images such as those showed in A–E. During the experiment, more than 600 virions in at least 10 different cells were evaluated for each sample. Data in the graph represent mean values ± s.d. from three independent experiments.

In non-treated control cells at 5 h p.i., virions co-localized with all of the tested markers (Figure 5A–E). Quantification of co-localization in control cells (Figure 5F, light grey bars) revealed that the highest percentage of virions co-localized with caveolin-1 (40.3±2.6%), but a substantial amount also co-localized with EGFP-Rab7 (26.5±2.9%) and small virus populations co-localized with EGFP-Rab11 (14.9±0.9%) and BiP (13.1±2.7%). Only residual co-localization of MPyV with early endosomal marker EGFP-Rab5 (5.9±1.6%) was detected at 5 h p.i. In the presence of microtubule-disrupting drug nocodazole (Figure 5F, dark grey bars), virus co-localization with caveolin-1 increased by 20% (to 48.4±2.9%), and a minor fraction of virions also co-localized with EGFP-Rab7 GTPase (13.2±2.5%). Only sporadic virions (∼2%) co-localized with other tested markers (EGFP-Rab5, EGFP-Rab11, BiP). In contrast, in cells with actin cytoskeleton disrupted by latrunculin A (Figure 5F, black bars), MPyV co-localization with caveolin-1 dropped by ∼50% (to 22.8±2.0%), while its co-localization with EGFP-Rab5 (8.0±2.4%), EGFP-Rab7 (40.1±4.1%), EGFP-Rab11 (20.1±2.7%) and BiP (18.3±5.2%) increased by 30–50% when compared to that in untreated cells. As the presence of the virions in early endosomes was sporadic even in control cells at 5 h p.i., we performed co-localization analysis earlier, at 1.5 h p.i., when a higher population of virions could be expected to co-localize with Rab5 GTPase. In untreated cells at that time p.i., a minor virus population co-localized with EGFP-Rab5 (12.2±2.2%). Virus co-localization with EGFP-Rab5 was markedly reduced in the presence of nocodazole (to 4.5±1.1%), and substantially increased (by 60%) in the presence of latrunculin A (to 19.5±3.6%) (Figure 5G).

These data demonstrate that disruption of microtubules by nocodazole significantly perturbed the virus presence in endosomes positive for Rab5, Rab7 and Rab11 GTPases and in the ER. The presence of latrunculin A apparently enhanced the efficiency of microtubule-mediated transport, and thus a higher percentage of virions co-localized with Rab5 and later post-infection with Rab7, Rab11 and BiP when compared to control untreated cells. We thus conclude that dynein-mediated transport along microtubules is important already for trafficking of MPyV from the plasma membrane to endosomes of classical endocytic pathways, while the actin meshwork rather represents a barrier that slows down the rate of virus endocytic transport and is connected with accumulation of the virions in caveolin-1-positive compartments.

### In the Absence of Microtubules, MPyV is Accumulated in Multicaveolar-like Clusters

**Figure 6:**
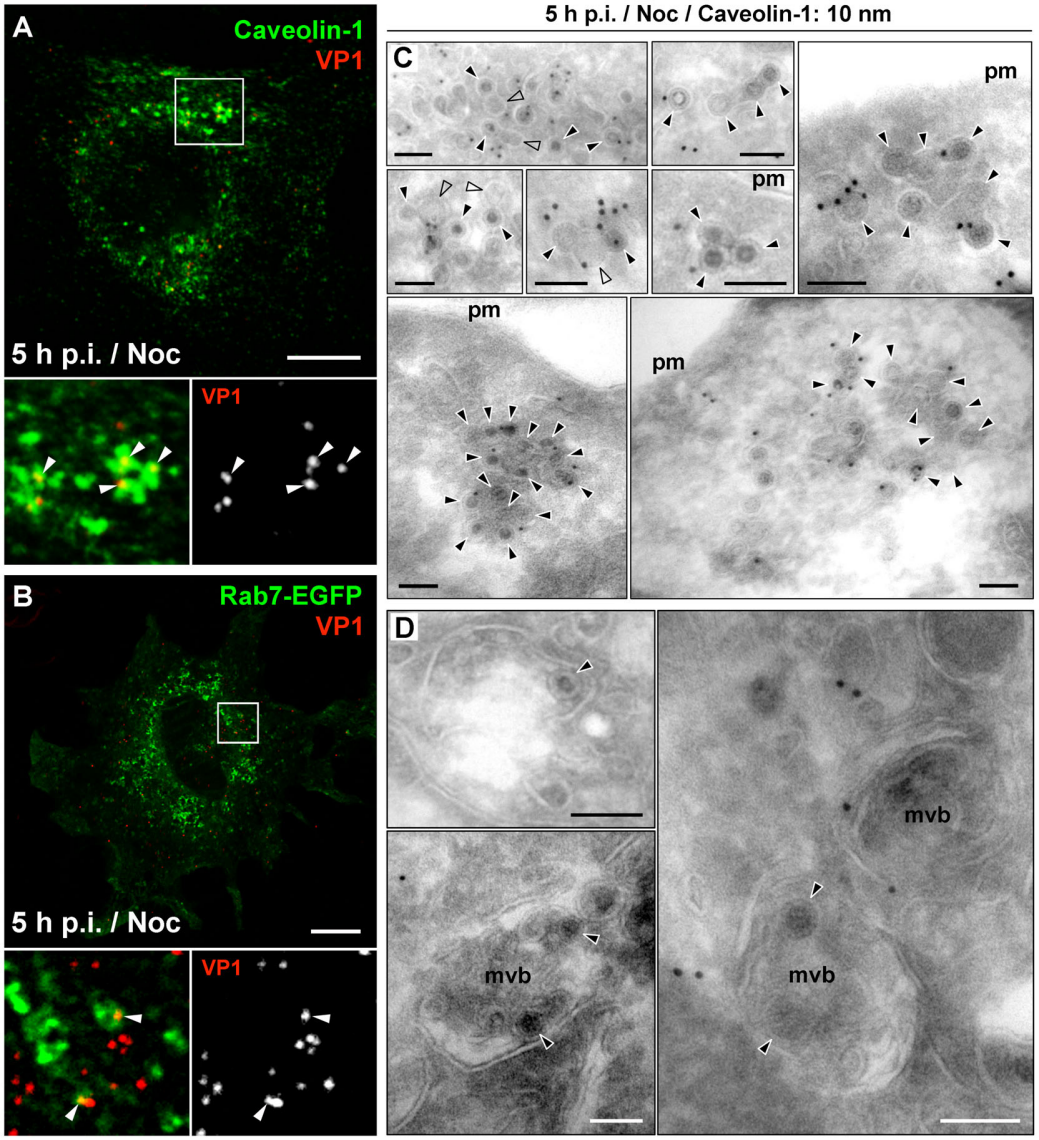
Localization of MPyV in nocodazole-treated cells. 3T6 cells were pre-treated (1 h) with nocodazole, infected with MPyV in the presence of the drug and fixed 5 h p.i. (A) Cells immunostained for MPyV VP1 (red) and caveolin-1 (green). (B) Cells expressing EGFP-fused Rab7 GTPase (green) immunostained for VP1 protein (red). Confocal sections of cells are shown. Arrowheads point to selected MPyV virions co-localizing with indicated marker. Bars, 10 µm. (C and D) Immunolabeling of thawed cryosections of cells with anti-caveolin-1 antibody, followed by immunolabeling with secondary antibody conjugated with 10 nm gold particles (seen as darkly stained dots). Arrowheads point to selected virions. Empty arrowheads point to flask-shape “empty” caveolar structures. Bars, 100 nm. Pm, plasma membrane; MVBs, multivesicular bodies.

**Figure 7:**
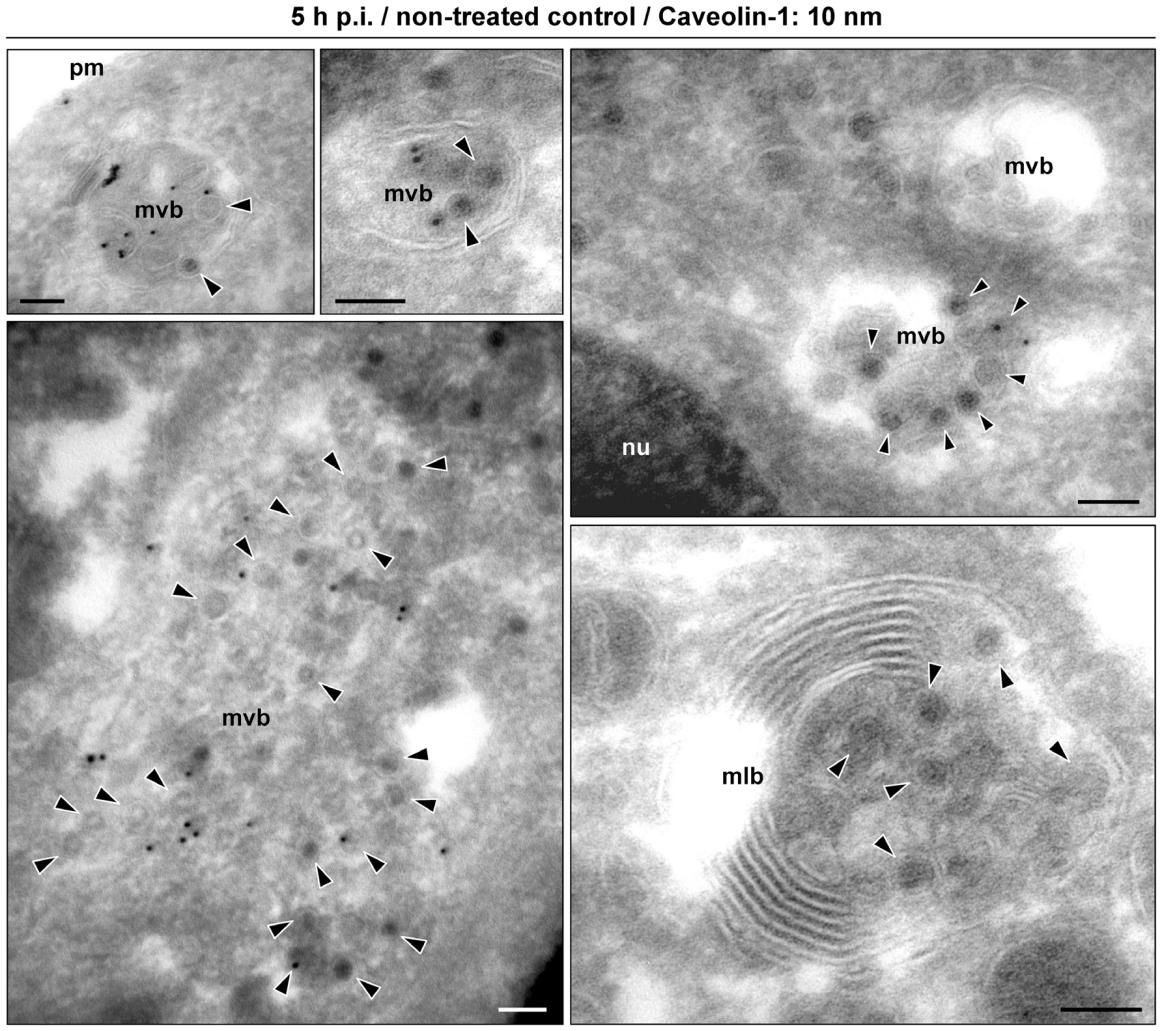
Immuno-electron microscopy of 3T6 cells at 5 h post-infection with MPyV. Thawed cryosections were immunolabeled with anti-caveolin-1 antibody followed by incubation with secondary antibody conjugated with 10 nm gold particles. Arrowheads point to selected virions. Bars, 100 nm. Pm, plasma membrane; MVBs, multivesicular bodies; MLB, multilamellar body; Nu, nucleus.

In the above analysis, only a minor subpopulation of the virus appeared in Rab7-positive endosomes of nocodazole-treated cells, while the amount of virions located within structures positive for caveolin-1 increased by 20% in comparison to the non-treated cells (Figure 6A,B, quantified in Figure 5F). To identify caveolin-1-positive compartments where MPyV virions appear in the absence of microtubules, we processed nocodazole-treated cells later (5 h) post-infection for immunogold labeling of caveolin-1 on thawed cryosections of the cells. Immunoelectron microscopy revealed that most virions were located at the cell periphery in tightly-fitting endocytic vesicles (∼60 nm in diameter), often also positive for caveolin-1. Virus-carrying vesicles were found connected to flask-shape caveolae-like empty structures (70–100 nm in diameter), but also to other virus-carrying vesicles, creating caveolin-1-positive membrane clusters of irregular shape (Figure 6C) whose morphology resembled multicaveolar complexes [43], [44]. Apart from these structures, only individual virions were found in late endosomal multivesicular bodies (MVBs), occasionally also positive for caveolin-1, and in caveolin-1-free endosomes (Figure 6D). These endosomal structures probably represented early or pre-mature late endosomes, since maturation of endosomes and their fusion with lysosomes is dependent on intact microtubular network and dynein motor function [45]–[48]. In contrast to nocodazole-treated cells, in non-treated cells at 5 h p.i. the accumulation of MPyV in caveolin-1-positive clusters was not apparent. Virions were found accumulated within multilamellar bodies or within MVBs also stained for caveolin-1 (Figure 7). This suggests that a high percentage of MPyV virions co-localizing with caveolin-1 in untreated cells (Figure 5F) is largely made up of virus within late endosomal structures. Together, these data demonstrate that in a situation when microtubular transport is not available for MPyV, most virions persist at the cell periphery within endocytic vesicles or within multicaveolar-like clusters.

**Figure 8:**
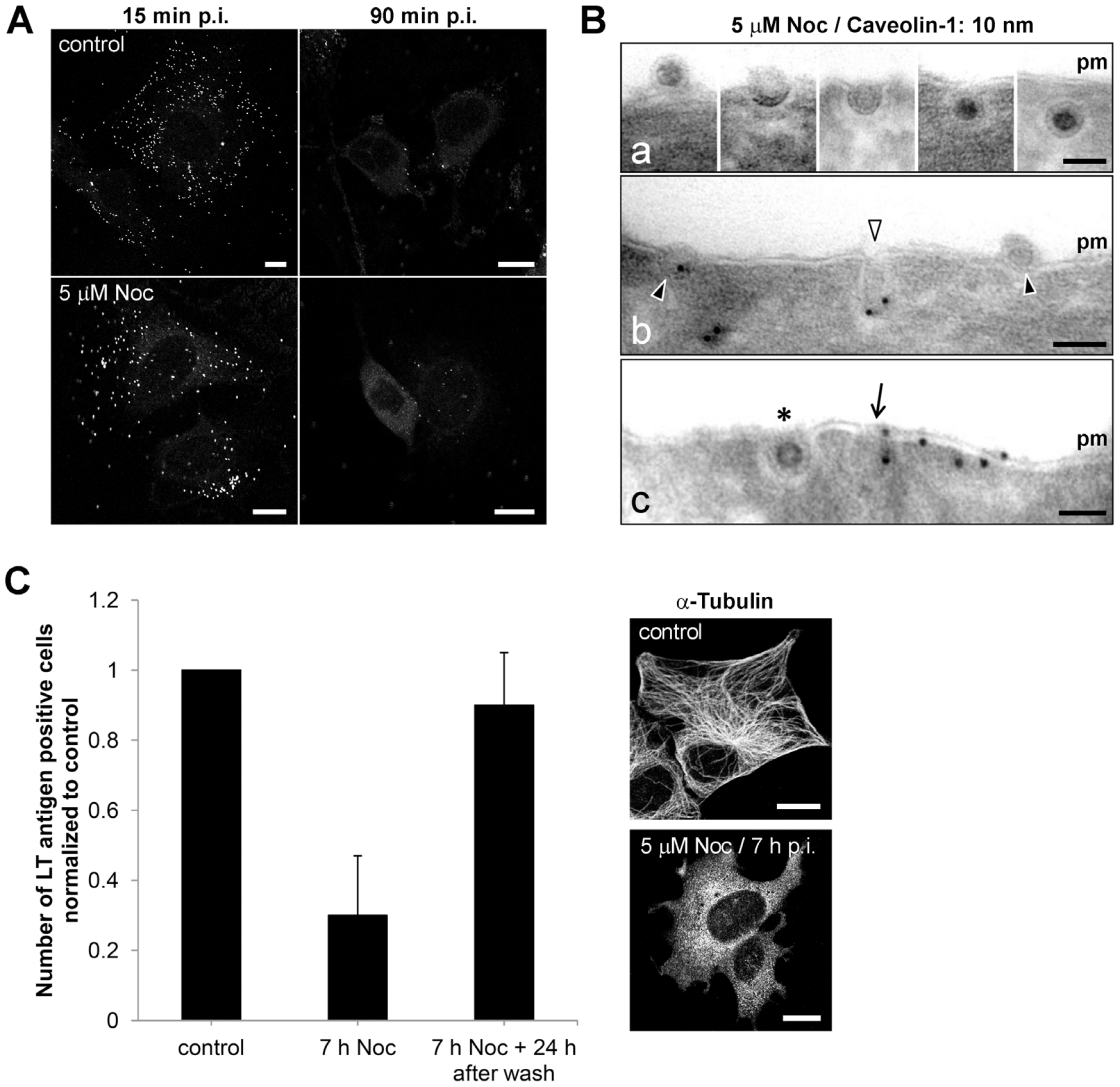
Internalization and infectivity of MPyV in nocodazole-treated cells. (A) 3T6 cells were pre-treated in culture medium alone or in medium supplemented with nocodazole for 1 h at 37°C and incubated with MPyV (MOI of 5×10^2^ virus particles/cell) for 15 or 90 min at 37°C, also in the presence or absence of the drug. After that, immunofluorescence analysis was performed using an anti-MPyV VP1 antibody added to live cells, followed by fixation. Confocal sections of representative cells are shown. Bars, 10 µm. (B) Immunolabeling of thawed cryosections of cells pre-treated and infected with MPyV in the presence of nocodazole. Cells were immunolabeled with anti-caveolin-1 antibody, followed by immunolabeling with secondary antibody conjugated with 10 nm gold particles (seen as darkly stained dots). Arrowheads point to selected virions. Empty arrowhead points to caveolar invagination. Asterisk indicates virion internalizing to an invagination lacking caveolin-1. Arrow points to virion internalizing via a region at the plasma membrane enriched for caveolin-1. Pm, plasma membrane. Bars, 50 nm. (C) 3T6 cells were pre-treated (1 h) with nocodazole and infected with MPyV. The drug was washed out at 7 h p.i. and cells were further incubated until 24 h p.i. (middle bar) or for additional 24 h after washing (right bar). As a control, cells were infected in the absence of the drug and fixed 24 h p.i. Cells were immunostained for MPyV LT antigen and the efficiency of infection was determined by the levels (%) of LT antigen-positive cells, normalized to that in control. During the experiment, at least 500 cells of each sample were counted. Data in the graph represent mean values ± s.d. from three independent experiments. Immunofluorescent staining of microtubules (anti-α-tubulin antibody; panel on the right) shows the morphology of microtubular network at the time of washing (7 h p.i.) in control or nocodazole-treated cells. Bars, 10 µm.

The resemblance of caveolin-1-positive clusters of MPyV-carrying vesicles in nocodazole-treated cells to multicaveolar membrane structures prompted us to test whether the presence of the drug affected the way of virus internalization, since previous studies provided evidence that internalization of MPyV is caveolae-independent [1]–[3], [6], [8]. To explore that, we first performed live-cell labeling of infected untreated or nocodazole-treated cells at 15 or 90 min p.i. with anti-MPyV VP1 primary antibody, to visualize non-internalized virions (see Materials and Methods). For both, untreated or nocodazole-treated cells, immunostaining at 15 min p.i. revealed abundant presence of MPyV virions attached to the cell surface, whereas at 90 min p.i., only residual presence of virus attached at the surface of cells was detected (Figure 8A). This observation suggests that virus uptake was not prevented by nocodazole. Further, we followed the way of MPyV internalization in the presence of nocodazole by immunoelectron microscopy. We found that virions are internalized independently of caveolar invaginations into tightly-fitting endocytic vesicles (Figure 8B, panels a and b) and that the presence of caveolin-1 on virus-carrying vesicles is caused by virus uptake via caveolin-1-rich domains at the plasma membrane (Figure 8B, panel c). These results indicate that the way of MPyV internalization is not affected by nocodazole. In addition, we tested the impact of temporary absence of a functional microtubular network on virus infectivity. We pre-treated (1 h prior to infection) and infected cells with MPyV in the presence of nocodazole. We found that more than 70% inhibition of MPyV infection in the cells kept with the drug until 7 h p.i. could be efficiently restored by the drug washout and by prolonged time of incubation (for additional 24 h) before cell fixation and screening (Figure 8C). Such reversibility supports our microscopic observations indicating that nocodazole does not affect MPyV entry and also points to the effective productive transport of virions when their accessibility to microtubules was recovered.

Together, these data indicate that in a situation when microtubular transport is not available, most of MPyV virions persist at the cell periphery within endocytic vesicles or within multicaveolar-like clusters, where they are inaccessible for surface labeling with antibody. Our results also suggest that the presence of virions in these structures does not affect their ability to infect cells and that with accessibility to microtubular network the virions continue in their trafficking to the endosomes and further to ER.

### Recycling Endosomes are not Required for MPyV Infection

**Figure 9:**
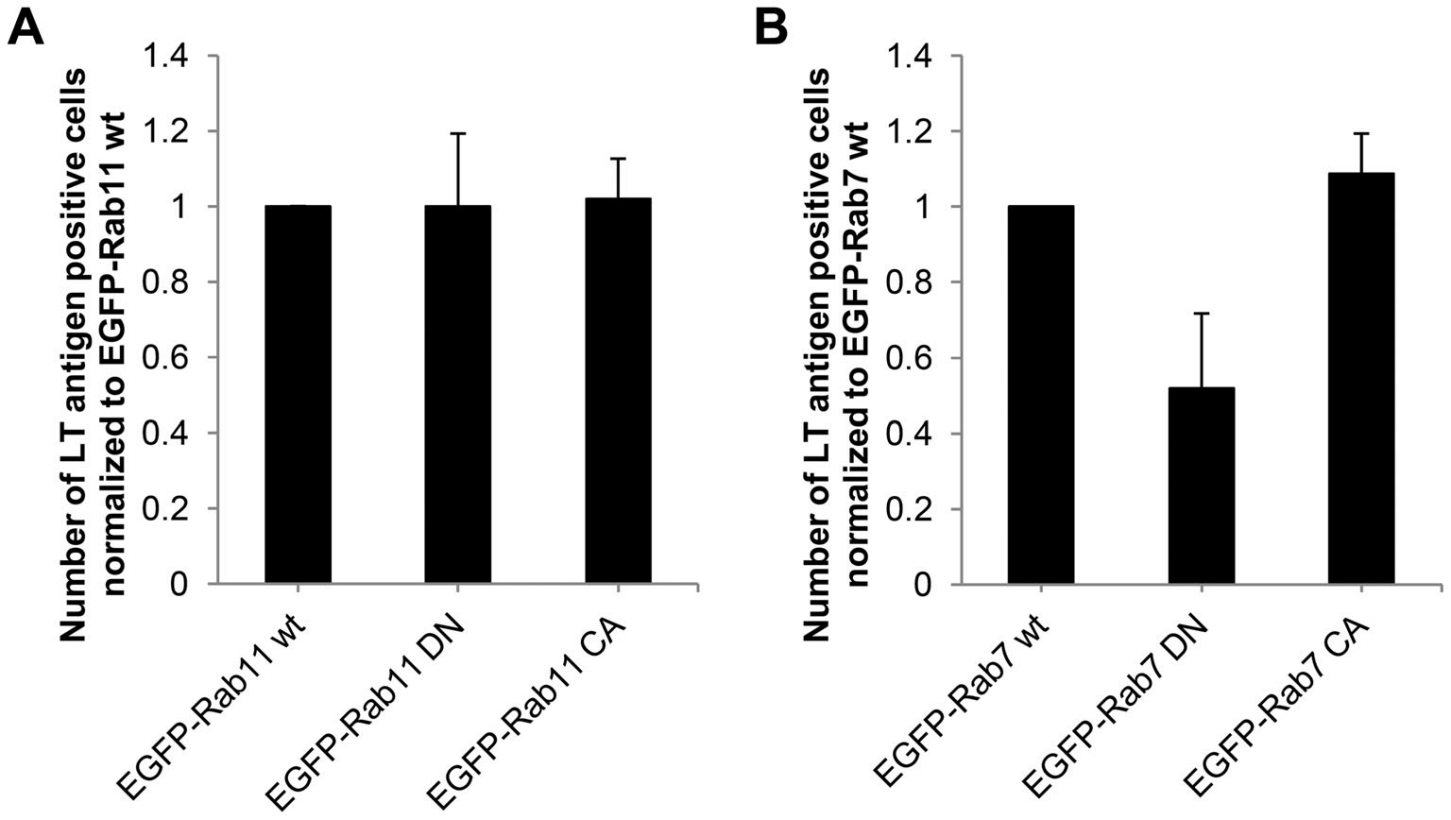
Rab11 GTPase is not required for MPyV infection. 3T6 cells were transfected with plasmid DNA for transient expression of (A) wild-type EGFP-Rab11 (wt), dominant-negative EGFP-Rab11 (DN) or constitutively active EGFP-Rab11 (CA), or (B) wild-type EGFP-Rab7 (wt), dominant-negative EGFP-Rab7 (DN) or constitutively active EGFP-Rab7 (CA). After 24 h, cells were infected with MPyV, incubated until 24 h p.i., fixed and immunostained for MPyV LT antigen. The efficiency of infection was determined by the levels (%) of LT antigen-positive cells from that expressing EGFP-fused version of the Rab11 or Rab7 GTPase normalized to that obtained in cells expressing its wild-type version. During the experiment, more than 500 cells were counted for each sample or control. Data in the graph represent mean values ± s.d. from three independent experiments.

In previous reports we showed that substantial amounts MPyV VP1 capsid protein appeared in Rab11-positive recycling endosomes of 3T6 cells [3], [10]. To investigate whether recycling endosomes participate in MPyV productive trafficking, we followed the virus infectivity in 3T6 cells transiently expressing EGFP-fused wild-type, dominant-negative or constitutively active mutant of Rab11 GTPase. As an additional control, we tested the virus infectivity during transient expression of analogous mutants of Rab7 GTPase, used previously to prove the dependence of MPyV infection on late endosomes in NIH 3T3 cells [9]. Our infection assays revealed that neither expression of EGFP-fused wild-type nor any mutant version of Rab11 GTPase affected the virus infectivity (Figure 9A). On the other hand, the expression of dominant-negative EGFP-Rab7 substantially reduced virus infectivity in comparison to the wild-type or constitutively active Rab7 version (Figure 9B). These data indicate that virus transport to the Rab11-positive recycling compartments is dispensable for MPyV. The functional expression of Rab11 GTPase constructs was confirmed by the intracellular distribution of fluorescently tagged transferrin. In accordance with observations of others [49], [50], in the cells expressing the wild-type or constitutively active version of Rab11, most of the transferrin was accumulated in EGFP-Rab11-positive structures concentrated in the juxtanuclear region corresponding to the pericentriolar recycling compartment, whereas in cells expressing the dominant-negative form, EGFP-Rab11 was dispersed in the cytoplasm or created thin tubular perinuclear elements, but the presence of transferrin in these structures was diminished (Figure S3).

## Discussion

In this study, we focused on the dynamics of intracellular transport of MPyV virions towards the nucleus and addressed the relevance of virus movements along microtubules for its productive infection. Here, we present evidence that dynein mediates all the critical steps of MPyV trafficking, including efficient virus transport from the plasma membrane to endosomes of classical endocytic pathways, maturation of MPyV-carrying endosomes, and subsequent virus delivery to the ER. In addition, we show that microtubule-dependent virus transport to recycling endosomes is not required for MPyV infection.

Previous studies that focused on the role of microtubular network in trafficking of polyomaviruses reported that perturbation of the dynein motor function did not lead to significant inhibition of JCV, SV40 or BKV infection [18], [20]. However, in accordance with our results, Engel et al [23] recently published RNA interference screening that revealed the dependence of SV40 infection on dynein motor in HeLa cells. The reason why some polyomaviruses do not require the dynein motor in some cell types is not clear. Ashok and Atwood [18] suggested that for transport in glial cells, JCV and SV40 require a different member of the dynein family whose function is independent of dynactin complex, or that the viral proteins can interact directly with microtubules for transport. The latter possibility can be ruled out in the case of MPyV, as the dynamics of long-distance movement of MPyV virions reflected microtubular motor processivity. Plus end-oriented movements of MPyV along microtubules mediated by kinesin-1 or kinesin-2 are apparently not required for virus productive trafficking. It has been found that kinesin-2 is required for normal steady-state localization of late endosomes [41]. However, despite the abnormal subcellular distribution of these compartments, authors of this study have shown that the uptake and trafficking of molecules through the conventional endocytic pathway was unaffected by inhibition of kinesin-2. Our results imply that this might also be true for trafficking of cargo via early endosomes whose motility requires kinesin-1 as well [51], since transfer via early endosomes is also important for MPyV [3], [9].

The requirement of minus end-oriented dynein motor observed for MPyV trafficking underlies the polyomavirus strategy how to reach the nucleus. Polyomaviruses do not target the nucleus directly via nuclear pore complexes but, still enclosed in vesicles or endosomes, they have first to reach the ER for uncoating the viral genomes [11]–[13], [52]. This strategy is in contrast with the one used by some enveloped viruses, e.g. HIV or Herpes viruses, which use bi-directional transport to reach the vicinity of the nucleus where they replicate. These viruses use dynein to reach the MTOC (microtubule organizing centre) and they can use kinesin-1 for their further transport from MTOC to the proximity of a nuclear envelope. However, capsids of these viruses, unlike those of polyomaviruses, travel usually naked in the cytosol, interacting directly with both, dynein and kinesin-1 motors (reviewed in [28]).

The results presented in this study indicate that utilization of the microtubular network and dynein motor by MPyV is different from that observed for SV40. We found that overexpression of dynamitin affected MPyV transport from the plasma membrane and reduced its presence in ER. Disruption of microtubules by nocodazole perturbed the presence of MPyV in classical endosomes, and only a small virus population was detected in premature late endosomes. Engel et al [23] observed no difference in the efficiency of SV40 trafficking to Rab5- or Rab7-positive endosomes in nocodazole-treated and non-treated cells. Nocodazole only blocked maturation of SV40-containing endosomes and subsequent transport of virions to ER. This group reported previously that vesicles carrying SV40 virions recruit actin in the form of actin comet tails and use it for their transport from the plasma membrane [25], suggesting that SV40 rather utilizes actin dynamics than the dynein motor to reach early and premature late endosomes. Although we have observed the involvement of actin dynamics in MPyV movement in 3T6 cells (Figure S2, Movie S3), disruption of actin microfilaments by latrunculin A did not prevent MPyV trafficking to classical endosomes. Moreover, it enhanced its efficiency. Correspondingly, virus infectivity in the presence of latrunculin A was higher than that in non-treated 3T6 cells (our unpublished results). Previous reports observing that treatment with actin-disrupting compounds increased MPyV infectivity in other naturally permissive cell lines are in agreement [2], [15].

Exploring localization of MPyV in the absence of functional microtubular transport in more detail revealed virus retention at the periphery of cells, within endocytic vesicles often connected to flask-shape caveolae-like empty structures, or within membrane clusters heavily labeled for caveolin-1. The morphology of virion-containing clusters strikingly resembled multicaveolar complexes described previously [43], [44]. These studies provided evidence that stimulation of caveolar endocytosis induces clustering of caveolae into multicaveolar complexes, mostly connected to the plasma membrane. However, we observed that upon nocodazole treatment, MPyV virions entered the cells independently of caveolae, similarly as described for untreated cells [3], [6], [8]. The presence of caveolin-1 on some virus-carrying vesicles is apparently caused by internalization of the virions via caveolin-rich domains at the plasma membrane (Figure 8B, panel c). Recently, formation of multicaveolar complexes was shown to occur along actin microfilaments from individual caveolar domains physically linked to the microfilament structure [53]. This supports our observation that the virus presence in caveolin-1-positive compartments was reduced by disruption of actin microfilaments (Figure 5F). We thus hypothesize that during internalization of MPyV via caveolin-rich domains at the plasma membrane, invaginating virions might be, due to the presence of caveolins, organized along actin microfilaments similarly to caveolar domains. Such virus internalizing invaginations might thus create their interconnections or connections with “empty” caveolae in their proximity. As no apparent difference in the detection of virions at the plasma membrane of nocodazole-treated or non-treated cells was observed 90 min p.i., we speculate that these clusters are intracellular or connected to the cell surface by very narrow invaginations. Interestingly, SV40 was detected by electron microscopy in similar membrane clusters, presented as structures of caveosomes [21]. Since caveosomes were later identified as late endosomes or lysosomes modified by overexpression of caveolin-1 [23], [26], we have to point out the presence of MPyV in structures whose morphology corresponds to the previous definition of caveosomes, but which by nature are not late endosomal compartments.

What is the fate of the virus in multicaveolar-like clusters? Trafficking of internalized caveolae into Rab11-positive endosomes has been reported [54]. We found transport of MPyV to Rab11-positive endosomes to be dependent on intact microtubules (Figure 5F), consistent with the finding that Rab11 together with the dynein motor mediate transport to the endosomal-recycling compartments [55]. For the virus, this apparently represents non-productive transport, as the expression of dominant-negative or constitutively active Rab11 GTPase did not affect MPyV infection. The appearance of MPyV in these compartments may thus be connected with recycling of the virus material back to the plasma membrane, as slow sorting of the VP1 signal from the perinuclear region to the cell periphery was observed in the interval 6–24 h p.i. (our unpublished results). However, the almost complete reversibility of inhibition of virus infection by nocodazole after its washout suggests that microtubules and dynein motor mediate the link for MPyV between multicaveolar-like structures and acidic endosomes. Several studies have reported caveolin-1 or caveolae trafficking into early endosomes [56] and, under specific conditions, also into MVBs and endolysosomal compartments [26], [43], [57] (reviewed in [58], [59]). Nevertheless, our results bring evidence that the presence of MPyV in multicaveolar-like complexes is dispensable for virus infection and rather decreases and/or delays productive trafficking of the MPyV virions.

## Materials and Methods

### Ethics Statement

*De novo* cell lines were established with approval by the Institutional Review Board of Faculty of Science, Charles University in Prague (approval number: 2012/06).

### Cell Line Cultivation and Transfection

Swiss albino mouse fibroblasts 3T6 (American Type Culture Collection, ATCC # CCL-96) were grown at 37°C and 5% CO_2_ in complete Dulbecco’s Modified Eagle’s Medium (DMEM) (Sigma-Aldrich, St Louis, MO, USA) supplemented with 10% fetal bovine serum (FBS) (Sigma-Aldrich) and GlutaMAX-I (Gibco, Life Technologies). All transfections were performed by electroporation using Amaxa kits (Lonza, Basel, Switzerland) according to the manufacturer’s instructions.

### DNA Constructs and Stable Cell Lines

For stable expression of EGFP-tagged α-tubulin or β-actin, cells were transfected with pEGFP-human α-Tub vector (Clontech, cat. 6117–1) or with pEGFP-human β-actin vector (Clontech, cat. 6116-1), respectively. Cell lines were established by sub-cloning and maintained upon G418 (Sigma-Aldrich) selection antibiotic in DMEM culture medium supplemented with 10% FBS and 4mM GlutaMAX-I. Plasmid for expression of dynamitin-EGFP (pDynamitin-EGFP-N1) was kindly provided by Beate Sodeik (MHH Institute of Virology, Hannover, Germany) [60]. Plasmid for expression of RFP-tagged dominant-negative kinesin-1: C-terminal fragment of kinesin-1 heavy chain (pRFP-KHCct) with control vector (pRFP-DTC) were kindly provided by Victoria J. Allan (University of Manchester, Manchester, United Kingdom) [39], [40]. Plasmids for expression of EGFP-fused dominant-negative subunits of kinesin-2: motorless KIF3A subunit (pEGFP-KIF3A-HL) and C-terminus of KAP3 accessory subunit (pEGFP-KAP3-CT) were kindly provided by Trina A. Schroer (The John Hopkins University, Baltimore, MD, USA) [41], [42]. Plasmids for expression of EGFP-fused wild-type [61], dominant-negative (S25N) and constitutively active (Q70L) Rab11 mutant were kindly provided by Marino Zerial (Max Planck Institute, Dresden, Germany). Plasmids for expression of EGFP-fused wild-type, dominant-negative (T22N) and constitutively active (Q67L) Rab7 mutant were kindly provided by Cecilia Bucci (The University of Salento, Lecce, Italy) [62]. Vector for expression of EGFP-fused wild-type version of Rab5 was kindly provided by Philip D. Stahl (Washington University School of Medicine) [63]. For the infection assays evaluating the efficiency of MPyV infection, vector pEGFP-N1 (Clontech) was used as a control.

### Virus

The A3 strain of MPyV (large-plaque strain) was isolated from infected WME (whole mouse embryo) cells according to Türler and Beard [64] and purified to homogeneity by CsCl and sucrose gradient ultracentrifugation. The quality of preparation was confirmed by Coomassie blue-stained sodium dodecyl sulfate-acrylamide gel electrophoresis and electron microscopy (negative staining). For microscopy of living cells, virions were labeled with the red fluorescent marker Alexa Fluor 546 carboxylic acid succinimidyl ester (Molecular Probes, Life Technologies) according to the manufacturer’s protocol as described in Liebl et al [3]. Viral titers were determined by plaque assays and particle numbers by hemagluttination assays. For infections, MPyV was used at the indicated multiplicities of infection (MOIs).

### Virus Tracking

For live microscopy, cells were grown in glass-bottom dishes (MatTek, Ashland, MA, USA) in phenol red-free DMEM culture medium supplemented with 10% FBS. Glass-bottom dishes were then mounted into a CO_2_-supplemented chamber, maintained at 37°C. To avoid rapid temperature changes and microtubule depolymerization at 4°C, all procedures were performed at 37°C with pre-warmed media and solutions. The fluorescently labeled virus was diluted in serum-free culture medium and added to cells at MOI of 10^2^ to 10^3^ particles per cell. Unbound virus was gently washed away after 20 min and complete culture medium was added. Cytoplasmic transport was monitored by time-lapse live imaging using a Leica TCS SP2 AOBS confocal microscope equipped with an Argon laser (458, 476, 488, 496, 514 nm; 10 mW) and a HeNe laser (543, 594 nm; 1 mW). Cells were examined with a 1.2 N.A. water immersion objective (60x). To minimize the possibility of tracking of not yet internalized virions attached to the cell surface, a complete z-scanning of the cell starting from the apical until the basal cell membrane was performed. After this step, the objective focus was fixed on the middle plane of the cell, and only virions moving in the internal area of the cell corresponding to the cytosol were tracked. According to the specific signal to noise ratio and EGFP level of expression, we applied different sampling frequencies (ΔT = 3–6 s). Sequential scanning between channels was used to separate fluorescence emission from different fluorochromes and to completely eliminate bleed through channels. EGFP-tubulin-expressing cells were alternatively examined with an Olympus IX81 CellR microscope equipped with an MT20 illumination system and a 63× oil-immersion objective, using Tx Red and GFP filter cube set. Video sequences and images were processed by Image J software (NIH, Bethesda, MD, USA) and Adobe Photoshop (Adobe Systems, San Jose CA, USA), respectively. Velocities and trajectories were calculated by ‘particle tracking’ plug-ins for Image J software (NIH) and data were processed with Excel software (Microsoft Corporation).

### Immunofluorescence Staining

For fixed-cell staining, cells were fixed with 4% formaldehyde in PBS (20 min) and permeabilized with 0.5% Triton X-100 in PBS (5 min). Alternatively, to follow the virus localization in EGFP-Rab7-positive compartments, we fixed cells with 4% formaldehyde plus 0.05% glutaraldehyde in PBS (60 min) as combined fixative was shown to sufficiently preserve the fragile structures of late endosomes for immunofluorescence analysis [62], [65]. After washing in PBS, cells were incubated with 0.25% bovine serum albumin and 0.25% porcine skin gelatin in PBS. Immunostaining with primary and secondary antibody was carried out for 1 h and 30 min, respectively, with extensive washing in PBS after each incubation. The following primary antibodies were used: rat monoclonal anti-MPyV large T (LT) antigen (kindly provided by B. E. Griffin, Imperial College of Science, Technology and Medicine at St. Mary’s, London, United Kingdom), mouse monoclonal anti-MPyV VP1 (prepared in our laboratory), rabbit polyclonal anti-MPyV VP1 (prepared in our laboratory), rabbit polyclonal anti-caveolin-1 (Santa Cruz, CA, USA), rabbit polyclonal anti-BiP (Abcam, Cambridge, UK), mouse monoclonal anti-α-tubulin (Exbio, Prague, Czech Republic) and rabbit polyclonal anti-GFP (Abcam). The following secondary antibodies were used: donkey anti-rat, donkey anti-mouse and goat anti-rabbit conjugated with Alexa Fluor 488; goat anti-rat conjugated with Alexa Fluor 546; Cy3-conjugated goat anti-mouse antibody (all purchased from Molecular Probes); and donkey anti-rabbit conjugated with CF633 fluorescent dye (Biotinum, CA, USA). DNA was stained with DAPI (4′, 6′-diamidino-2-phenylindole). For testing the EGFP-fused versions of Rab11 GTPase, transferrin tagged with Alexa Fluor 647 (Molecular Probes) was used.

Live-cell labeling was performed as described in Zhou et al [66]. At indicated times (15 or 90 min) post-infection, cells were washed three times with complete Dulbecco’s PBS (DPBS) at 37°C and incubated with mouse monoclonal anti-MPyV VP1 antibody for 20 min at 37°C. The cells were then washed three times with DPBS (37°C) and fixed with 4% formaldehyde in PBS (10 min). Fixed but not permeabilized cells were washed in PBS and incubated with Cy3-conjugated anti-mouse secondary antibody as described above.

### Internalization Assay

Cells grown on 13-mm glass coverslips in 24-well plates were incubated with MPyV labeled with Alexa Fluor 546 dye, diluted in serum-free culture medium and added to cells at MOI of 10^3^ particles per cell. After 20 min (at 37°C), cells were washed to remove unbound virus and complete DMEM medium (37°C) was added. Cells were incubated until indicated time points p.i. and fixed with 4% formaldehyde in PBS (20 min). The detection of non-internalized virions was accomplished by surface labeling of non-permabilized cells, washed in PBS and incubated with anti-MPyV VP1 antibody, followed by incubation with secondary antibody conjugated to green dye Alexa Fluor 488. The percentages of internalized virions were determined from the maximum-intensity projections of Z-stacks of confocal optical sections of the examined cells.

### Infectivity Assays

To determine MPyV infectivity during the expression of fluorescently tagged versions of the proteins or fluorescent proteins alone, cells were transfected with protein encoding plasmid DNA and seeded on 13-mm glass coverslips in 24-well plates. Cells were allowed to grow for 24 or 48 h (two days for expression of kinesin-2 subunits, as they turn out slowly [41]), washed and incubated with MPyV diluted in serum-free medium at MOI of 0.3 PFU/cell, for 1 h at 37°C. The infection start was measured from virus addition to cells. After virus adsorption, cells were washed to remove the unbound virus and incubated in DMEM with 10% FBS until 24 h p.i. and fixed. Fixed cells were immunostained with antibody against MPyV early LT antigen. The efficiency of infection was determined from the percentage of LT-positive cells from that expressing the fluorescently tagged protein of interest, normalized to that obtained in control cells.

To determine the reversibility of MPyV infection after nocodazole treatment, cells were pre-treated in medium with 5 µM nocodazole (Calbiochem, Merck) for 1 h at 37°C and in the presence of drug infected with MPyV as above (the functional disruption of microtubules by nocodazole was confirmed by immunostaining with anti-α-tubulin antibody). At 7 h p.i., the drug was washed out and the cells were incubated until 24 h p.i., or in parallel samples, cells were incubated for additional 24 h prior to fixation and immunostaining to prove the reversibility of the inhibition effect. Fixed cells were immunostained for the MPyV LT antigen and the cell nuclei were visualized by DAPI. The efficiency of infection was determined from the percentage of cells positive for LT antigen normalized to that obtained in cells infected in the absence of drug and fixed 24 h p.i.

For assay quantification, coverslips were observed with an Olympus BX-60 fluorescence microscope equipped with a COHU CCD camera and images of optical fields were taken using Lucia software (Laboratory Imaging, Prague, Czech Republic). Cells were counted using the ‘cell counter’ plug-in for Image J software (NIH).

### Quantification of Co-localization

Cells transfected with plasmid DNA for expression of the EGFP-fused protein of interest, mock-transfected or non-transfected cells were seeded on 13-mm coverslips in 24-well plates and left to grow for 16–24 h. Cells at 10–20% of confluency were used. For co-localization analysis in the presence of cytoskeleton drugs, cells were pre-incubated in culture medium alone (control) or medium containing 5 µM nocodazole or 0.1 µM latrunculin A (Calbiochem, Merck) for 1 h (at 37°C) prior to infection (the functional disruption of microtubules or microfilaments by the drugs was confirmed in parallel samples by immunostaining with anti-α-tubulin antibody or with rhodamine-conjugated phalloidin, respectively). Cells were infected with MPyV diluted in serum-free culture medium (with or without the drug) at MOI ∼5×10^2^ virus particles/cell, allowing quantification of individual virions (counted approximately from 30 to 120 virions per cell). The infection start was measured from virus addition to cells. After 1 h at 37°C, the inoculum was removed, cells were washed three times to remove unbound virions, and complete DMEM medium +10% FBS (with or without drug) was added. Cells were incubated until indicated time p.i., fixed, and immunostained for MPyV VP1 capsid protein and the second marker of interest if not fused to EGFP. The cells were examined with a Leica TCS SP2 AOBS confocal microscope using a ×63 1.4 N.A. oil immersion objective. For each examined cell, Z-sections were taken and co-localization of individual virions was determined in individual sections using the ‘colocalization highlighter’ plug-in for Image J software (NIH). The intensity ratio of co-localized pixels was set at 50%. The obtained image with co-localizing pixels was merged with the image with MPyV VP1 signal and co-localized and non-colocalized virions were counted.

### Immunoelectron Microscopy of Cryosections

Cells grown on Petri dishes were pre-treated (1 h/37°C) in culture medium alone or medium containing 5 µM nocodazole and in the absence or presence of the drug were infected with MPyV at MOI of 5×10^3^ virus particles per cell for 1 h (rocking). Cells were washed and complete DMEM with 10% FBS (37°C) with or without nocodazole was added. Cells were fixed 5 h p.i. with 4% formaldehyde plus 0.05% glutaraldehyde solution in 0.1M Sörensen buffer (Na_2_HPO_4_/NaH_2_PO_4_, pH 7.2). Fixed cells were gently scraped from the plate with a rubber policeman and embedded in 10% gelatin in PBS. Gelatin-embedded samples were cut into 1-mm^3^ blocks and infiltrated with 2.3M sucrose overnight at 4°C. Infiltrated blocks were mounted onto aluminum pins, frozen in liquid nitrogen and then sectioned at −120°C with a Leica EM FC7 microtome. Ultrathin cryosections were transferred in a drop of 2.3M sucrose, 2% methylcellulose onto formwar-carbon-coated nickel grids and immunolabeled with rabbit polyclonal antibody against caveolin-1 (BD Transduction Laboratories, BD Biosciences), followed by incubation with goat anti-rabbit antibody conjugated to 10 nm gold particles (British Biocell Int.). After labeling, the sections were contrasted and embedded in a mixture of 3% aqueous uranyl acetate and 2% methylcellulose. The samples were observed with a JEM-1011 JEOL electron microscope equipped with a side mounted 2k×2k CCD Camera (Veleta, Olympus SIS).

## Supporting Information

Figure S1 **Internalization assay.** 3T6 cells were incubated with MPyV labeled with red fluorescent dye Alexa Fluor 546 (AF546-MPyV) diluted in serum-free medium (MOI of 10^3^ virus particles/cell) for 20 min at 37°C. After virus adsorption, cells were washed and incubated in complete DMEM medium (37°C) until indicated times p.i. The extracellular and intracellular AF546-MPyV virions (red) were distinguished by surface immunolabeling of fixed but not permeabilized cells with anti-MPyV VP1 antibody, followed by incubation with secondary antibody conjugated to green dye Alexa Fluor 488. (A and B) Visualization of AF546-MPyV virions (red) in permeabilized (A) and non-permeabilized (B) cells by immunostaining with anti-VP1 antibody (green). Confocal sections of cells fixed 60 min p.i. with enlarged details are shown. In panel B, arrows point to selected extracellular virions. (C) Quantification of the amount of internalized AF546-MPyV virions at 20, 30, 60 and 120 min p.i. The percentages of internalized virus were calculated from images such as shown in panel B. More than 1100 virions were evaluated for each time point. Data in the graph represent mean values ± s.d. for 10 different cells.
(TIF)

Figure S2 **Movement of MPyV-carrying endosomes associated with dynamic actin assemblies.** 3T6 cells stably expressing EGFP-fused β-actin (green) were infected with Alexa Fluor 546-labeled MPyV (red) (MOI of 10^2^ to 10^3^ virus particles per cell) at 37°C and scanned with ΔT = 4 s. Selected frames of cell at 45 min p.i. with corresponding transmission light images illustrate short-distance movement of virus-carrying endosomes associated with dynamic assemblies of EGFP-actin (see Movie S3). White arrowheads point to MPyV virions. Arrows point to endosome-associated actin assemblies. Black arrowheads indicate MPyV-containing endosomes. Bars, 5 µm. Cells were examined using a Leica TCS SP2 AOBS confocal microscope.
(TIF)

Figure S3 **Intracellular distribution of fluorescently tagged transferrin during expression of Rab11 GTPase mutants.** 3T6 cells expressing EGFP-fused wt, DN or CA version of Rab11 were incubated for 5 min (pulse) at 37°C with 25 µg/ml Alexa Fluor 647-transferrin. Cells were further incubated for 30 min (chase) at 37°C in serum-containing medium, fixed and processed for fluorescence microscopy. Confocal sections showing representative distribution of transferrin in the cells are presented. Arrows point to places of concentrated transferrin. Arrowheads point to tubular perinuclear elements of Rab11 DN. Bars, 10 µm.
(TIF)

Movie S1 **Bi-directional transport of MPyV virions along microtubules.** 3T6 cells expressing EGFP-tubulin (green) were infected with fluorescently labeled MPyV (red) at 37°C and analyzed by time-lapse fluorescence microscopy. Virions were transported in both directions: towards the nucleus (left) and to the cell periphery (right). Images were taken with intervals of 3 seconds. Video sequences were obtained using an Olympus IX81 CellR microscope.
(AVI)

Movie S2 **MPyV virions accumulate in perinuclear space later post-infection (3 h p.i.).** 3T6 cells expressing EGFP-tubulin (green) were infected with fluorescently labeled MPyV (AF594-MPyV; red) with MOI of 10^3^ virus particles per cell at 37°C and analyzed by time-lapse confocal microscopy. Images were taken with intervals of 6 seconds. Bar, 10 µm. Video sequence was obtained using a Leica TCS SP2 AOBS confocal microscope.
(AVI)

Movie S3 **Movement of MPyV associated with dynamic actin.** 3T6 cells expressing EGFP-actin (green) were infected with fluorescently labeled MPyV (red) at 37°C and analyzed by time-lapse confocal microscopy. Video sequence with fluorescent signals (left) and corresponding sequence from transmission light (right) are shown. Images were taken with intervals of 4 seconds. Video sequences were obtained using a Leica TCS SP2 AOBS confocal microscope.
(AVI)
