# Supplementary figures and images for: Involvement of Microtubular Network and Its Motors in Productive Endocytic Trafficking of Mouse Polyomavirus
Source: PLoS One. 2014 May 8;9(5):e96922. doi: 10.1371/journal.pone.0096922 (PMC4014599; doi:10.1371/journal.pone.0096922)

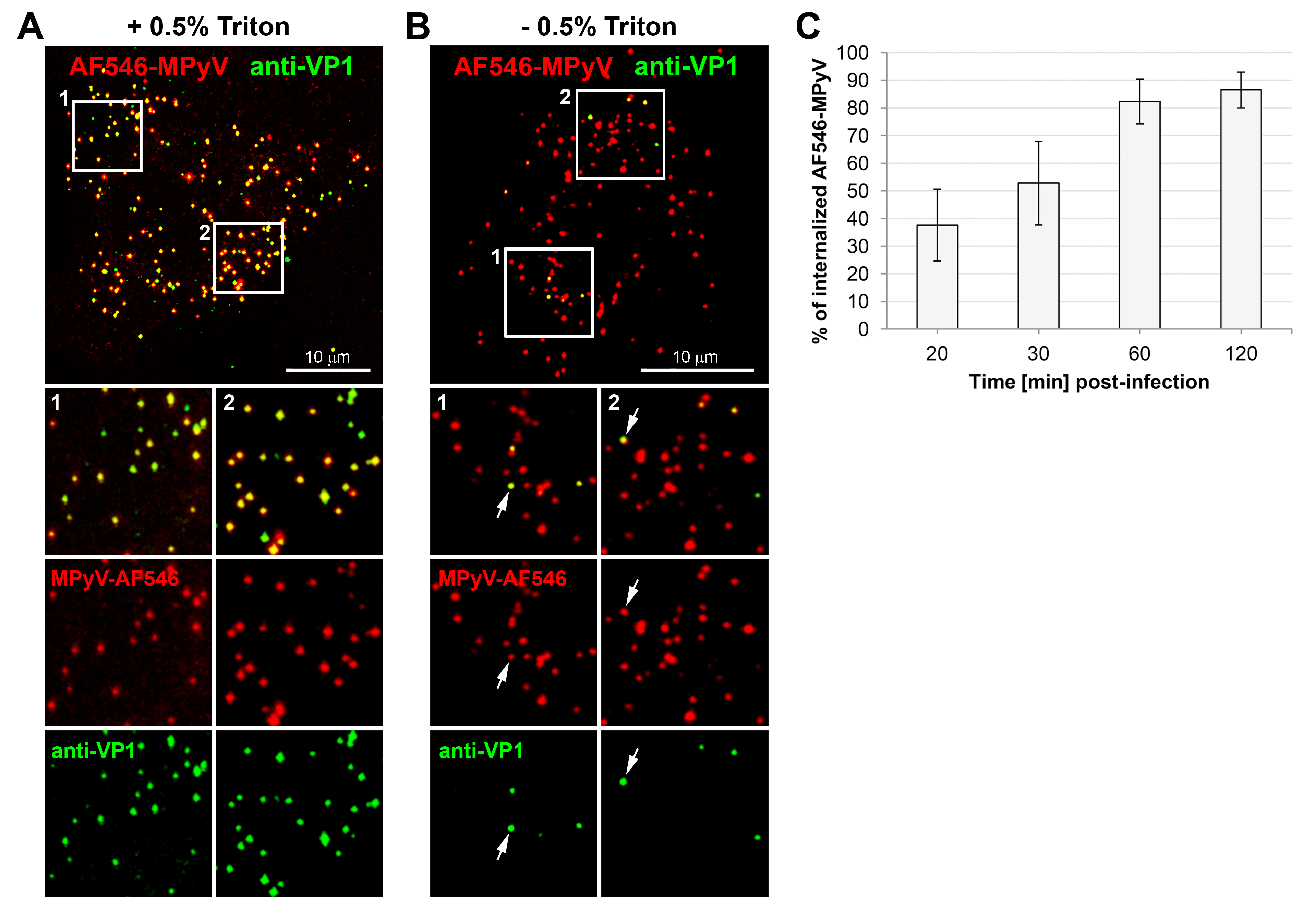

Supplement: Figure S1 — Internalization assay. 3T6 cells were incubated with MPyV labeled with red fluorescent dye Alexa Fluor 546 (AF546-MPyV) diluted in serum-free medium (MOI of 103 virus particles/cell) for 20 min at 37°C. After virus adsorption, cells were washed and incubated in complete DMEM medium (37°C) until indicated times p.i. The extracellular and intracellular AF546-MPyV virions (red) were distinguished by surface immunolabeling of fixed but not permeabilized cells with anti-MPyV VP1 antibody, followed by incubation with secondary antibody conjugated to green dye Alexa Fluor 488. (A and B) Visualization of AF546-MPyV virions (red) in permeabilized (A) and non-permeabilized (B) cells by immunostaining with anti-VP1 antibody (green). Confocal sections of cells fixed 60 min p.i. with enlarged details are shown. In panel B, arrows point to selected extracellular virions. (C) Quantification of the amount of internalized AF546-MPyV virions at 20, 30, 60 and 120 min p.i. The percentages of internalized virus were calculated from images such as shown in panel B. More than 1100 virions were evaluated for each time point. Data in the graph represent mean values ± s.d. for 10 different cells. (TIF) [file pone.0096922.s001.tif]

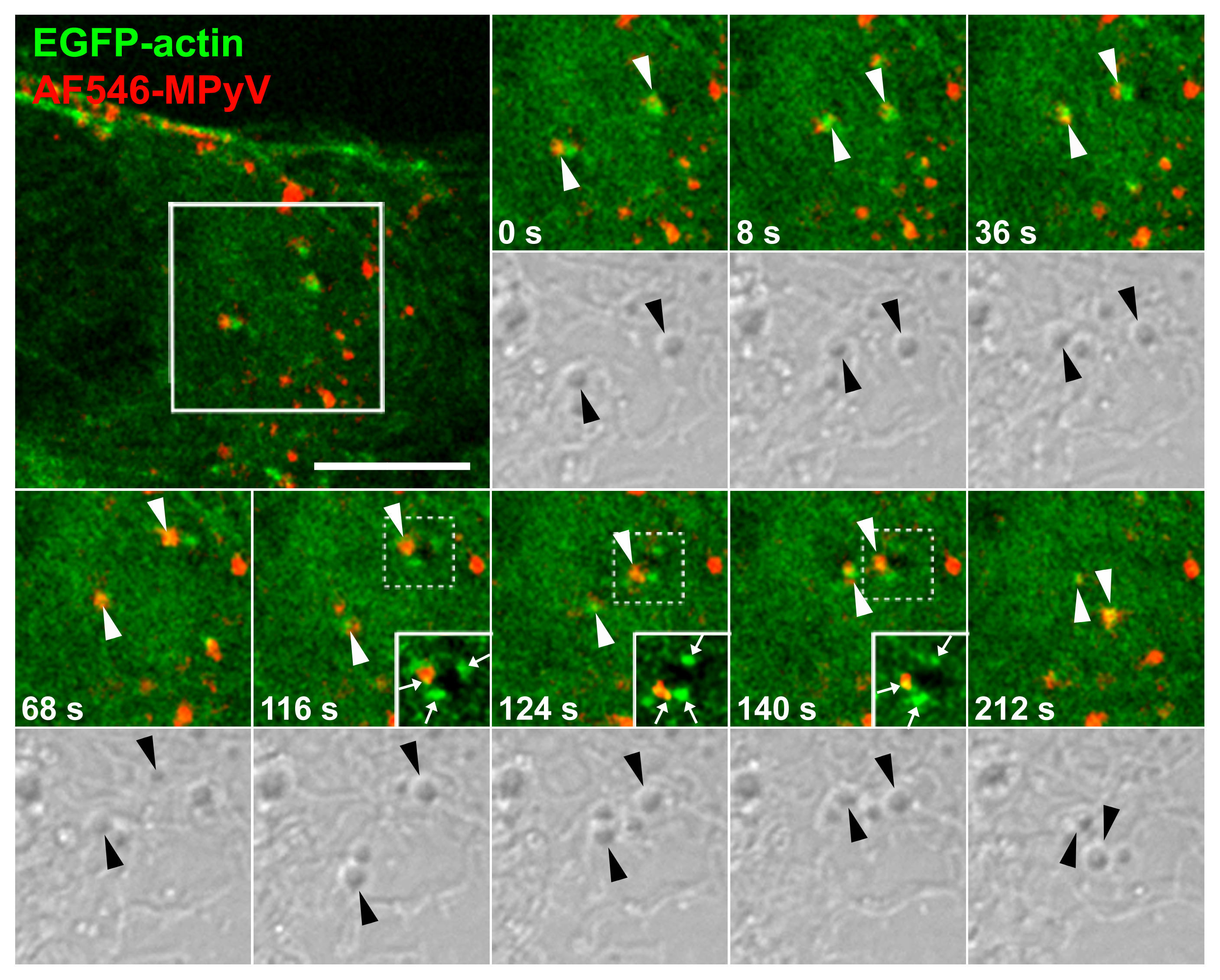

Supplement: Figure S2 — Movement of MPyV-carrying endosomes associated with dynamic actin assemblies. 3T6 cells stably expressing EGFP-fused β-actin (green) were infected with Alexa Fluor 546-labeled MPyV (red) (MOI of 102 to 103 virus particles per cell) at 37°C and scanned with ΔT = 4 s. Selected frames of cell at 45 min p.i. with corresponding transmission light images illustrate short-distance movement of virus-carrying endosomes associated with dynamic assemblies of EGFP-actin (see Movie S3). White arrowheads point to MPyV virions. Arrows point to endosome-associated actin assemblies. Black arrowheads indicate MPyV-containing endosomes. Bars, 5 µm. Cells were examined using a Leica TCS SP2 AOBS confocal microscope. (TIF) [file pone.0096922.s002.tif]

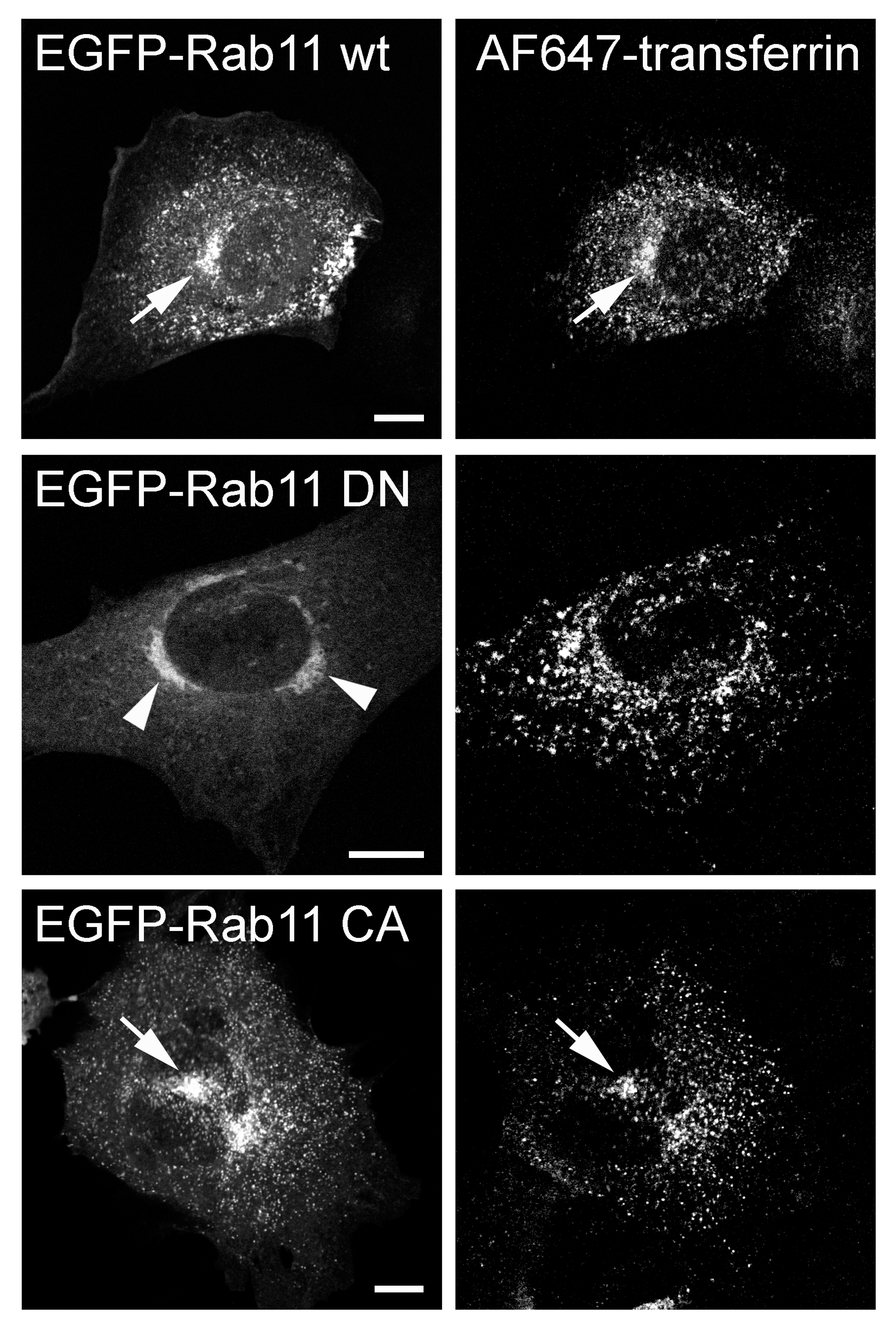

Supplement: Figure S3 — Intracellular distribution of fluorescently tagged transferrin during expression of Rab11 GTPase mutants. 3T6 cells expressing EGFP-fused wt, DN or CA version of Rab11 were incubated for 5 min (pulse) at 37°C with 25 µg/ml Alexa Fluor 647-transferrin. Cells were further incubated for 30 min (chase) at 37°C in serum-containing medium, fixed and processed for fluorescence microscopy. Confocal sections showing representative distribution of transferrin in the cells are presented. Arrows point to places of concentrated transferrin. Arrowheads point to tubular perinuclear elements of Rab11 DN. Bars, 10 µm. (TIF) [file pone.0096922.s003.tif]
